# Psychophysiology of duration estimation in experienced mindfulness meditators and matched controls

**DOI:** 10.3389/fpsyg.2015.01215

**Published:** 2015-08-18

**Authors:** Simone Otten, Eva Schötz, Marc Wittmann, Niko Kohls, Stefan Schmidt, Karin Meissner

**Affiliations:** ^1^Meissner Lab, Institute of Medical Psychology, Ludwig-Maximilians-UniversityMunich, Germany; ^2^Institute for Frontier Areas of Psychology and Mental HealthFreiburg, Germany; ^3^Division Integrative Health Promotion, University of Applied Sciences and ArtsCoburg, Germany; ^4^Department of Psychosomatic Medicine, University Medical Center FreiburgFreiburg, Germany; ^5^Institute for Transcultural Health Studies, European University ViadrinaFrankfurt (Oder), Germany

**Keywords:** mindfulness meditation, time perception, interoceptive awareness, autonomic regulation, attention

## Abstract

Recent research suggests that bodily signals and interoception are strongly related to our sense of time. Mindfulness meditators train to be aware of their body states and therefore could be more accurate at interval timing. In this study, *n* = 22 experienced mindfulness meditators and *n* = 22 matched controls performed both, an acoustic and a visual duration reproduction task of 8, 14, and 20 s intervals, while heart rate and skin conductance were continuously assessed. In addition, participants accomplished a heart beat perception task and two selective attention tasks. Results revealed no differences between meditators and controls with respect to performance in duration reproduction or attentional capacities. Additionally no group difference in heart beat perception scores was found. Across all subjects, correlational analyses revealed several associations between performance in the duration reproduction tasks and psychophysiological changes, the latter being also related to heart beat perception scores. Furthermore, former findings of linearly increasing cardiac periods and decreasing skin conductance levels during the auditory duration estimation task (Meissner and Wittmann, 2011) could be replicated, and these changes could also be observed during a visual duration reproduction task. In contrast to our earlier findings, the heart beat perception test was not related with timing performance. Overall, although experienced meditators did not differ from matched controls with respect to duration reproduction and interoceptive awareness, this study adds significantly to the emerging view that time perception is related to autonomic regulation and awareness of body states.

## Introduction

The diversity of psychological and neurophysiological models of time perception is indicative of the fact that the neural substrates and the processes accounting for the experience of time are still unknown (Wittmann and Paulus, 2009; Merchant et al., 2013). Recent conceptualizations that are based on empirical findings hint on the relationship between the experience of time and self–and body processes, that is, on interoceptive states (Craig, 2009, 2015; Wittmann, 2009, 2013): Recordings in functional magnetic resonance imaging (fMRI) indicated that activity within the insular cortex increased continuously during the perception of duration (Wittmann et al., 2010, 2011). Moreover, the experience of temporal delay between external acoustic stimulation and the heart beat is related to anterior insula activation (Critchley et al., 2004). Given the close connection between the insular cortex and interoceptive processes, the integration and perception of ascending bodily signals would substantially contribute to the experience of time.

Recordings of physiological signals (heart periods, skin conductance levels, respiratory periods) during a duration reproduction task, with intervals of 8, 14, and 20 s, showed not only a linear increase of cardiac periods during the encoding of temporal intervals, but also revealed a positive relationship between the slope of this increase in cardiac periods and duration reproduction accuracy (Meissner and Wittmann, 2011). A recent study also reported a positive correlation between a greater control of the vagal tone and duration reproduction accuracy for intervals from 0.5 to 40 s (Pollatos et al., 2014). That is, the increase in cardiac (parasympathetic) tone, as registered in the brain, could represent a bodily marker for the estimation of duration. It is important to mention that it is not the mean heart rate during a target interval that correlated with timing behavior (as assessed by Schwarz et al., 2013) but the linear increase of cardiac periods that showed this relationship. In addition, results of Meissner and Wittmann (2011) indicated that duration reproduction accuracy correlated positively with the subjects' ability to accurately perceive their own heart beats. A possible explanation would be that individuals, who have a greater awareness of their body states and thus a better access to visceral feedback, are more accurate at interval timing. Alternatively, the correlations between duration reproduction accuracy and both, the slope of cardiac slowing and interoceptive awareness could be due to increased attentional capacities of good performers in time estimation tasks, who then simply would be able to focus more adequately on time perception. In this scenario, the steeper slope of cardiac periods in good performers would reflect their higher attentional load, and the relationship with interoceptive awareness could be due to the recently reported association between interoceptive awareness and attention to external acoustic or visual stimuli that are used for presentation of temporal intervals (Matthias et al., 2009).

In this study, we investigated the role of body signals, interoceptive awareness, and attentional capacities for interval timing by focusing on a group of participants who regularly train their attentional capacities to internal and external stimuli, namely experts in mindfulness meditation. Mindfulness is understood as bringing awareness to each present moment in time with an accepting and non-judgmental attitude. It can be developed and cultivated through introspective training such as meditation (Kabat-Zinn, 2005; Sauer et al., 2013). Mindfulness meditators during a typical meditation session focus on their breathing-in and breathing-out or perform a “body scan” where attention is systematically directed to each area of the body from the toes to the top of the head (Morone et al., 2008). Effects of mindfulness meditation are manifold but basically comprise increased body awareness, attention regulation, and emotion regulation (Hölzel et al., 2011; Tang et al., 2015) all of which are mental factors that are involved in time perception (Wittmann and Schmidt, 2014). Accordingly, regular mindfulness meditation has been shown to alter the cortical representation of interoceptive attention (Farb et al., 2013). Moreover, there is evidence indicating that mindfulness meditators after intensive practice have increased attentional capacities to external stimuli (Jha et al., 2007; Zeidan et al., 2010; Moore et al., 2012). These findings suggest that mindfulness meditators may process time differently than matched controls. Indeed, mindfulness meditators report that subjective time slows down during mindfulness-oriented meditation and in daily life (Kabat-Zinn, 2005).

In this study, we aimed to compare the role of bodily signals and attentional capacities to internal and external stimuli for time estimation in *n* = 22 experienced mindfulness meditators and *n* = 22 matched controls. Participants performed two duration reproduction tasks, while heart rate and skin conductance were monitored. In addition, they accomplished a heart beat perception task and two selective attention tasks. We hypothesized that in comparison to non-meditating controls, the mindfulness meditation experts would reproduce time intervals more accurately. We also expected that mindfulness meditators would show higher attentional capacities and would score higher in interoceptive awareness. Finally, we investigated whether the expected differences between meditators and controls would go along with alterations of bodily signals during the duration estimation tasks (Meissner and Wittmann, 2011; Wittmann and Schmidt, 2014).

A secondary aim of the study was to investigate whether we could replicate our former findings of linearly increasing cardiac periods and decreasing skin conductance levels during the auditory duration estimation task (Meissner and Wittmann, 2011), and whether these changes could also be observed during an otherwise similar visual duration reproduction task.

## Materials and methods

### Design

We conducted a cross-sectional study comparing *n* = 22 experienced mindfulness meditators with *n* = 22 matched controls. The study was conducted at the Institute of Medical Psychology, University of Munich, as part of a larger two-center study, which compared 42 mindfulness meditators with 42 matched controls in their ability of time perception using a broad range of behavioral tasks (Wittmann et al., 2015). The study was approved by the ethical committees of the partaking Universities (Munich and Freiburg).

### Participants

Twenty-two experienced mindfulness meditation practitioners with at least three years of continuous practice and at least 2 h of practice a week over the last 8 weeks were recruited, as well as *n* = 22 matched controls without any meditation experience. The matching criteria were sex, age (± 3 years) and education (± 1 level of 5). Participants were recruited by advertisements in meditation centers, on online platforms of the universities, and by word of mouth. Meditators were included when reporting to regularly practice a form of meditation, which had a dominant orientation toward awareness of the present moment. Therefore, we included individuals if they participated in forms of mindfulness meditation, Vipassana meditation, or Soto Zen. Control subjects were required to have no experience with any form of meditation including Yoga or Tai-Chi. Age was restricted to the range between 21 and 50 years in order to constrain age-related effects in the psychophysical tasks and psychophysiological measurements. Participants had to be fluent in German and were additionally required to report good health and no known medical or psychological problems as assessed with a detailed screening questionnaire. All participants signed an informed consent and received a moderate financial compensation (€20) for participation.

### Instruments

#### Heart beat perception task

The heart beat perception task consists of four heart beat counting intervals (35, 25, 45, and 60 s), during which participants are asked to attend to their own heart beats and count them silently (Pollatos et al., 2007). The beginning and end of each counting interval is signaled by a start and stop tone. A heart beat perception score is calculated for each participant across the four trials according to the following equation where high scores (maximum = 1) indicate accurate heart beat perception:

$$\begin{array}{l} 1-1∕4\text{Σ}(\left(\left|\text{ recorded heart beats—counted heart beats}\right|\right)∕\\ \text{recorded heart beats}) \end{array}$$

#### Freiburg mindfulness inventory (FMI)

The FMI (Walach et al., 2006; Kohls et al., 2009) contains 14 items which evaluate self-reported mindfulness on the basis of a two-dimensional structure utilizing a 4-point item scale format. These dimensions are “presence” as ability to attend to the present moment (“I am open to the experience of the present moment”) and “acceptance” as non-judgmental attitude (“I am patient with myself when things go wrong”).

#### The attention network test (ANT)

The ANT (Fan et al., 2002) assesses the processing efficiency of the three attention networks of (1) alerting, (2) orienting, and (3) executive attention (conflict effect). They are quantified by means of computerized reaction time measures for differently cued and un-cued stimulus conditions. Participants have to respond to either the left or right arrow key on the computer keyboard depending on stimulus configuration. An overall reaction time score and an index of accuracy are also calculated.

#### Divided attention

Divided attention was assessed by a subtest of the “Test Battery for Attentional Performance” [TAP, Version 1.7;Psytest: Herzogenrath (Zimmermann and Fimm, 1997, 2002)]. The subtest comprised a dual task paradigm in which the participants were required to simultaneously monitor visual and acoustic stimuli. In the visual task participants had to detect whether crosses that appeared in a random configuration in a 4 × 4 matrix form the corners of a square. The acoustical task included a regular sequence of high and low beeps, whereas participants had to detect an irregularity in the sequence of these beeps.

#### Visual and auditory duration reproduction

In two separate computer tasks running on Psychtoolbox for Matlab subjects had to reproduce the duration of (1) a visual and (2) an auditory stimulus with intervals of 8, 14, and 20 s duration. In each trial, a green square (resp. a sinus tone of 440 Hz) was first presented for one of the three durations. After the pause of either 4.5, 5, or 5.5 s duration a second yellow square (resp. a sinus tone of 500 Hz) appeared. It had to be stopped by pressing the space bar when participants felt that this second stimulus had reached the duration of the first. The reproduction task contained six presentations of each of the three durations summing up to 18 trials. At the beginning and the end of each stimulus, an onset resp. offset marker signal was sent from the Matlab program to the physiological acquisition software. Participants were requested not to use mental strategies such as inner counting but to rely on their subjective feeling of elapsed time. To further prevent mental counting, participants were additionally given a secondary working-memory task. Before each trial four numbers were presented. At the end of each trial one number appeared and the subjects responded whether the number was one of the four previously presented by pressing the left or right arrow button, respectively for “yes” and “no.” The accuracy of duration reproduction can be calculated from the temporal reproduction performance. In the following, the mean of the six reproduced time intervals of each of the three durations is referred to as “reproduced duration.” A duration reproduction score (referred to as “duration reproduction accuracy”) was calculated for each participant and each of the three durations from six trials according to the following equation:

$$\begin{array}{l} 1-1∕6\text{Σ}(\left(\left|\text{presented duration—reproduced duration}\right|\right)∕\\ \text{presented duration}) \end{array}$$

Similar to the heart beat perception score high scores (maximum 1) indicate accurate duration reproduction.

### Physiological recordings

Heart rate and electrodermal activity were continuously recorded using a BIOPAC MP 150 device (BIOPAC Systems Inc., Goleta, CA, USA) with AcqKnowledge 4.1 software for data acquisition. The sampling rate was 500 Hz for the ECG signal and 15.625 Hz for the electrodermal signal.

Heart rate was measured using three disposable Ag/AgCl electrodes which were positioned in an Einthoven Lead I configuration and connected to the BIOPAC amplifier module ECG100C. Skin conductance was measured using two disposable Ag/AgCl electrodes which were attached to the thenar and hypothenar portions of the left hand and connected to the BIOPAC amplifier module GSR100C.

### Data reduction and statistical analysis

Intervals between successive R peaks (cardiac periods) were extracted from the electrocardiogram signal using the peak-detection function implemented in AcqKnowledge 4.2. Cardiac periods were examined and screened for artifacts based on the procedure developed by Porges and Byrne (1992). Cardiac periods were re-sampled at 15.625 Hz by using cubic interpolation. Cardiac periods and skin conductance levels were averaged for every second across the six presentations of each of the three durations (8, 14, 20 s). Skin conductance levels were log-transformed prior to statistical analysis to obtain normal distributions.

Differences between study groups where analyzed by Student-*t*-tests for normally distributed data, Mann–Whitney *U*-tests for non-parametric data, and Chi-Quadrat tests for categorial data. Group differences for performance in the auditory and visual duration reproduction tasks were explored by mixed-design ANOVAs, with the between-subjects factor “group” (mindfulness meditators, controls) and the within-subjects factor “interval” (8, 14, 20 s). To evaluate changes over time in cardiac periods and skin conductance levels separate mixed-design ANOVAs were performed for the encoding and reproduction phase of each duration interval. Due to individual differences in the length of the individually reproduced intervals, the analyses for physiological changes during the reproduction intervals for auditory and visual reproduction tasks were restricted to seconds that were available for all subjects. In the auditory reproduction task we analyzed the first 6, 8, and 12 s of the 8, 14, and 20 s intervals in the reproduction phase of the task, respectively. In the visual reproduction tasks the analyses were restricted to the first 6, 8, and 15 s for cardiac periods and 6, 8, 12 s for skin conductance levels of the 8, 14, and 20 s intervals in the reproduction phase of the task, respectively. Greenhouse-Geisser corrections for repeated measures ANOVAs were applied where appropriate.

Individual linear slopes of cardiac periods and skin conductance levels for each of the 18 encoding intervals were determined using linear regression. In order to normalize the data, individual time series were converted into z-scores before performing the linear regression analyses. Individual slopes were then averaged for each participant across the six presentations for each of the three durations. Group differences between slopes were explored by mixed-design ANOVAs with “interval” as the within-subjects factor and “group” as between-subjects factor.

Spearman-Rho's correlations were used to explore relationships between behavioral results in the duration reproduction tasks, associated psychophysiological changes, heart beat perception scores, and results in the attention tests.

For statistical tests the significance level was set to *p* ≤ 0.05. Whenever multiple tests were conducted, the significance level was Bonferroni-corrected.

## Results

### Study sample

On average, the mindfulness meditators had a meditation experience of 10.4 years (± 7.5 SD) and had practiced 7 h per week (± 5 SD). Meditation and control groups did not differ with respect to age, education, body mass index, physical activity, or current stress levels. As expected, meditators showed significantly higher values of self-reported mindfulness in the “Freiburg Mindfulness Inventory” (FMI) in both subscales of “presence” and “acceptance,” as well as in the sum score (Table 1).

**Table 1:** **Characteristics of the two study groups**.

**Variable**	**Mindfulness meditators (*n* = 22)**	**Matched controls (*n* = 22)**	***p*-value^a^**
Age (mean ± SD)	39.7 ± 7.9	39.5 ± 8.0	0.953^b^
Sex (female (%))	12 (55)	12 (55)	0.619^c^
Body mass index (mean ± SD)	21.6 ± 2.3	21.9 ± 2.7	0.675
Educational level			0.794^c^
Primary school; *Hauptschule*, n (%)	0	1 (5)	
Secondary modern school; *Realschule*, n (%)	1 (5)	1 (5)	
Grammer school*; Gymnasium*, n (%)	4 (18)	4 (18)	
University or college degree; *Studium*, n (%)	17 (77)	16 (73)	
Physical Activity (ln IPAQ, mean ± SD)	7.84 ± 0.65	7.76 ± 1.10	0.778
Current stress level (SQCB, mean ± SD)	12.73 ± 3.7	12.45 ± 4.5	0.822
Mindfulness (FMI)			
Presence (mean ± SD)	25.2 ± 2.8	20.3 ± 3.9	**<0.001**
Acceptance (mean ± SD)	19.3 ± 2.1	16.4 ± 37.5	**0.001**
Sum score (mean ± SD)	44.7 ± 4.2	37.5 ± 5.8	**<0.001**

*IPAQ, International Physical Activity Questionnaire; SQCB, Short Questionnaire on Current Burden; FMI, Freiburg Mindfulness Inventory*.

*Bold values signify p < 0.05*.

a *t-Test if not otherwise indicated*.

b *Mann–Whitney U-Test*.

c *Chi-Square Test*.

### Heart beat perception scores

**Figure 1:**
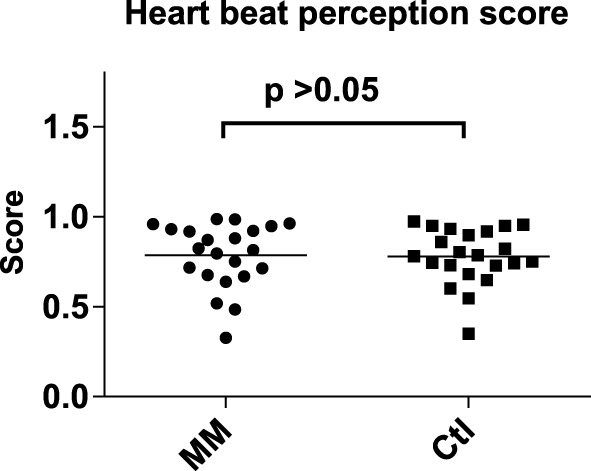
**Statistical dot plot with means (black bars) showing heart beat perception scores in mindfulness meditators (MM) and matched controls (Ctl)**. Mann–Whitney *U*-Tests were used to evaluate group differences.

The mean heart beat perception scores were 0.79 (± 0.18 SD) in meditators and 0.78 (± 0.15 SD) in controls, with no significant difference in-between (Mann–Whitney *U*-test, *p* = 0.751; Figure 1).

### Attentional capacities

**Figure 2:**
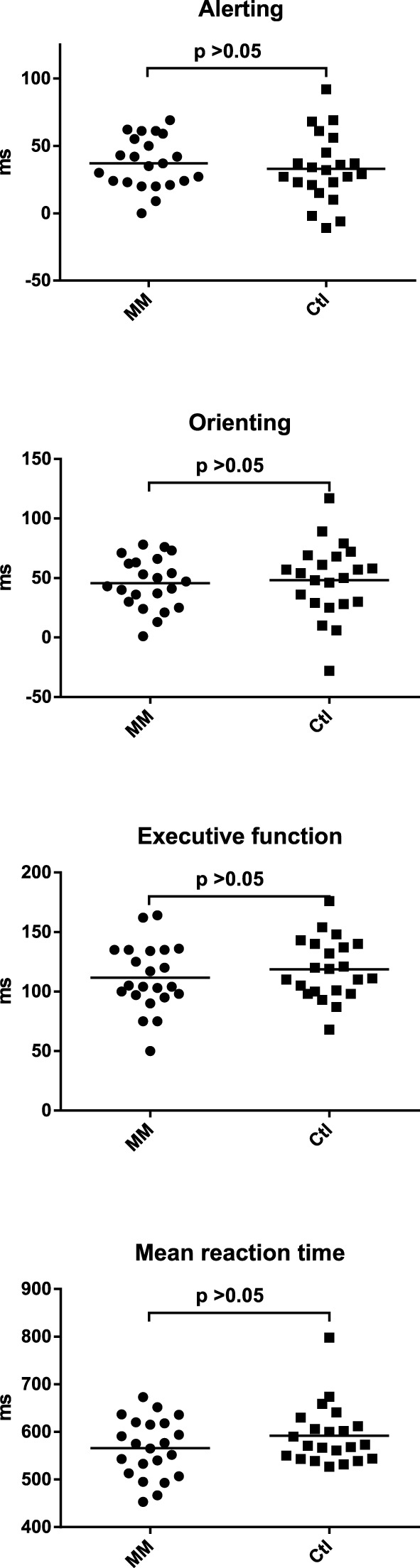
**Statistical dot plots with means (black bars) showing the results of the Attention Network Test (ANT) in mindfulness meditators (MM) and matched controls (Ctl)**. *T*-Tests were used to evaluate group differences.

Meditators and controls did not differ in any of the variables of the Attention Network Test (Figure 2; Supplementary Table 1) or in the mean accuracy for the TAP subtest “divided attention” (Supplementary Table 1).

### Duration reproduction tasks

**Figure 3:**
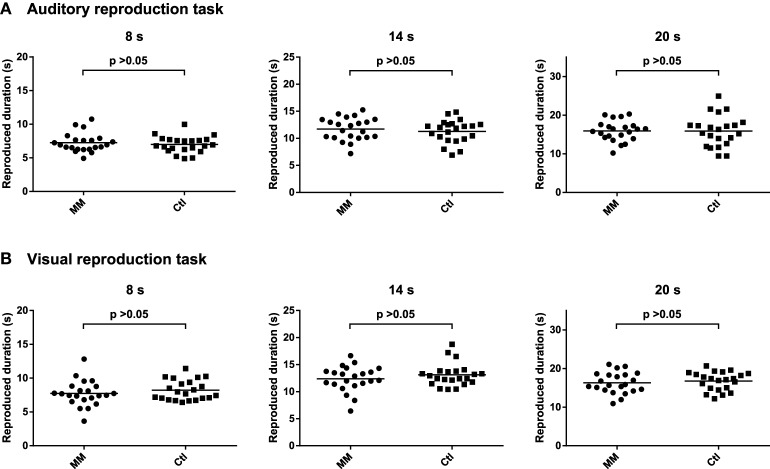
**Statistical dot plots with means (black bars) showing reproduced duration for the three intervals of the auditory (A) and visual (B) duration reproduction tasks in mindfulness meditators (MM) and matched controls (Ctl)**. Mixed analyses of variance revealed no significant differences between groups [main effect of group, auditory task: *F*_(1, 42)_= 0.4, *p* = 0.514; visual task: *F*_(1, 42)_ = 0.9, *p* = 0.345]. *P*-values correspond to the results of simple effects *T*-tests.

Neither in the auditory nor in the visual duration reproduction tasks reproduced duration differed significantly between mindfulness meditators and matched controls (Figure 3; Supplementary Table 2). The respective analyses for the accuracy of duration reproduction also did not reveal any significant group differences (Supplementary Table 2).

### Psychophysiological changes in duration reproduction tasks

#### Cardiac periods

**Figure 4:**
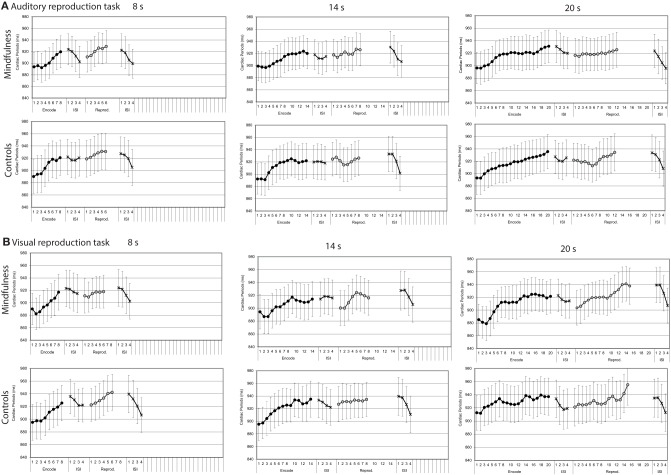
**Cardiac periods by groups (mindfulness meditators, controls)**. Mean second-to-second changes of cardiac periods (ms) during the encoding intervals, the reproduction intervals, and first 4 s of the subsequent inter-stimulus intervals (ISI) for tones of 8, 14, and 20 s duration of both groups (for statistical results, see Table 2). **(A)** Auditory reproduction task. **(B)** Visual reproduction task. Error bars represent standard error of the mean.

In both the auditory and visual reproduction tasks mean cardiac periods increased over time during the encoding intervals of 8, 14, and 20 s duration and decreased during the first 4 s after the tone had stopped (Figure 4). Mixed-design ANOVAs showed significant main effects for time for each of the three encoding intervals of 8, 14, and 20 s duration during both the auditory and the visual tasks, and linear trends fitted the data reasonably well; no significant main effects for group or interaction effects for time by group were revealed (Table 2).

**Table 2:** **Results of the mixed-design ANOVAs for cardiac periods during the three encoding and reproduction intervals of the auditory and visual duration reproduction tasks**.

	**Interval**	**Main effect for time**		**Linear trend**		**Interaction effect (time by group)**		**Main effect for group**	
		***F*[df, error(df)]**	***p***	***F*[df, error(df)]**	***p***	***F*[df, error(df)]**	***p***	***F*[df, error(df)]**	***p***
Auditory Task	**AUDITORY ENCODING INTERVAL**								
	8 s	16.38 (2.46, 103.28)	<0.001^**^	32.15 (1, 42)	<0.001^**^	1.15 (2.46, 103.28)	0.328	0.009 (1, 42)	0.926
	14 s	15.96 (4.74, 199.10)	<0.001^**^	74.21 (1, 42)	<0.001^**^	0.99 (4.74, 199.10)	0.423	0.002 (1, 42)	0.961
	20 s	13.25 (5.78, 242.93)	<0.001^**^	59.33 (1, 42)	<0.001^**^	0.40 (5.78, 242.93)	0.875	0.000 (1, 42)	0.999
	**AUDITORY REPRODUCTION INTERVAL**								
	8 s	4.83 (1.93, 80.92)	0.011^**^	6.66 (1, 42)	0.013^**^	0.22 (1.926, 80.912)	0.797	0.018 (1, 42)	0.893
	14 s	1.43 (3.57, 150.10)	0.233	1.06 (1, 42)	0.307	1.49 (3.57, 150.10)	0.212	0.002 (1, 42)	0.962
	20 s	2.44 (4.22, 177.05)	0.045^*^	5.40 (1, 42)	0.025^*^	0.65 (4.22, 177.05)	0.633	0.006 (1, 42)	0.939
Visual Task	**VISUAL ENCODING INTERVAL**								
	8 s	15.07 (2.523, 100.92)	<0.001^**^	26.28 (1, 40)	<0.001^**^	0.33 (2.523, 100.92)	0.770	0.08 (1, 40)	0.779
	14 s	14.73 (4.48, 179.34)	<0.001^**^	63.07 (1, 40)	<0.001^**^	0.99 (4.48, 179.34)	0.421	0.17 (1, 40)	0.680
	20 s	14.23 (5.22, 208.75)	<0.001^**^	41.03 (1, 40)	<0.001^**^	2.50 (5.22, 208.75)	0.029^*^	0.26 (1, 40)	0.614
	**VISUAL REPRODUCTION INTERVAL**								
	8 s	4.91 (2.33, 93.26)	0.007^**^	8.25 (1, 40)	0.006^**^	1.05 (2.33, 93.26)	0.363	0.22 (1, 40)	0.639
	14 s	3.66 (2.88, 115.14)	0.146	5.56 (1, 40)	0.023^*^	1.84 (2.88, 115.14)	0.146	0.26 (1, 40)	0.638
	20 s	8.44 (4.89, 195.49)	<0.001^**^	38.88 (1, 40)	<0.001^**^	1.24 (4.89, 195.49)	0.292	0.05 (1, 40)	0.828

* *p < 0.05*.

** *p < 0.0167 (Bonferroni-corrected level of significance)*.

*Bold values signify p < 0.05*.

Compared with the respective encoding intervals, cardiac periods showed similar patterns during the reproduction intervals of the respective tasks (Figure 4). Mixed-design ANOVAs confirmed some of the changes to be significant, but the results were more ambiguous than during the encoding intervals (Table 2).

#### Skin conductance levels

**Figure 5:**
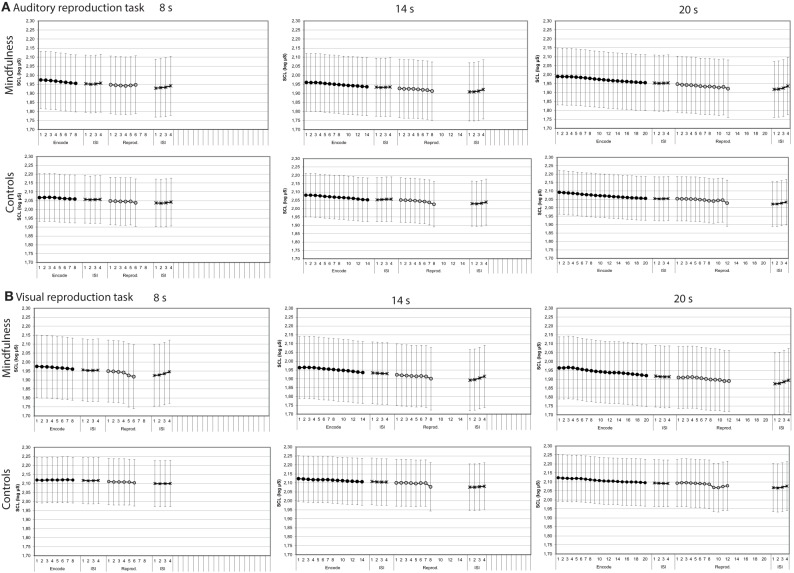
**Skin conductance levels by groups (mindfulness meditators, controls)**. Mean second-to-second changes of skin conductance levels (log μS) during the encoding interval, the reproduction interval, and first 4 s of the subsequent inter-stimulus intervals (ISI) for tones of 8, 14, and 20 s duration of both groups (for statistical results, see Table 3). **(A)** Auditory reproduction task. **(B)** Visual reproduction task. Error bars represent standard error of the mean.

Both in the auditory and visual reproduction tasks, mean skin conductance levels decreased over time during the encoding intervals of 8, 14, and 20 s duration and increased during the first 4 s after the tone had stopped (Figure 5). Mixed-design ANOVAs showed significant main effects for time for each of the three encoding intervals of 8, 14, and 20 s duration in the auditory task, and linear trends fitted the data reasonably well (Table 3). In the visual duration reproduction task main effects for time were significant for the encoding intervals of 14 and 20 s duration. No significant main effects for group were found in neither task. A significant interaction effect group by time was revealed during the 8 s-encoding interval of the visual reproduction task [*F* = 4.90 (1.62, 63.23); *p* = 0.0163], in that meditators showed a sharper decline of skin conductance levels than controls (Figure 5; Table 3).

**Table 3:** **Results of the mixed-design ANOVAs for skin conductance levels during the three encoding intervals of the auditory and visual duration reproduction tasks**.

	**Interval**	**Main effect for time**		**Linear trend**		**Interaction effect (time by group)**		**Main effect for group**	
		***F*[df, error(df)]**	***p***	***F*[df, error(df)]**	***p***	***F*[df, error(df)]**	***p***	***F*[df, error(df)]**	***p***
Auditory Task	**AUDITORY ENCODING INTERVAL**								
	8 s	22.01 (1.62, 63.08)	<0.001^**^	27.92 (1, 39)	<0.001^**^	2.46 (1.62, 63.08)	0.104	0.26 (1, 39)	0.638
	14 s	42.39 (1.55, 60.52)	<0.001^**^	54.42 (1, 39)	<0.001^**^	0.27 (1.55, 60.52)	0.706	0.33 (1, 39)	0.571
	20 s	29.38 (1.38, 53.75)	<0.001^**^	35.69 (1, 39)	<0.001^**^	0.15 (1.38, 53.75)	0.780	0.24 (1, 39)	0.631
	**AUDITORY REPRODUCTION INTERVAL**								
	8 s	0.29 (1.19, 46.38)	0.632	0.39 (1, 39)	0.536	0.42 (1.19, 46.34)	0.557	0.23 (1, 39)	0.634
	14 s	4.40 (1.14, 44.57)	0.037^*^	7.02 (1, 39)	0.012^**^	0.39 (1.14, 44.57)	0.561	0.34 (1, 39)	0.565
	20 s	1.80 (1.19, 46.30)	0.186	5.11 (1, 39)	0.029^*^	0.08 (1.19, 46.30)	0.826	0.29 (1, 39)	0.594
Visual task	**VISUAL ENCODING INTERVAL**								
	8 s	3.56 (1.62, 63.23)	0.043^*^	4.27 (1, 39)	0.045^*^	4.90 (1.62, 63.23)	0.016^**^	0.477 (1, 39)	0.494
	14 s	22.45 (1.72, 67.11)	<0.001^**^	29.75 (1, 39)	<0.001^**^	2.68 (1.72, 67.11)	0.084	0.543 (1, 39)	0.446
	20 s	35.12 (2.33, 91.00)	<0.001^**^	62.40 (1, 39)	<0.001^**^	2.18 (2.33, 91.00)	0.110	0.567 (1, 39)	0.456
	**VISUAL REPRODUCTION INTERVAL**								
	8 s	3.65 (1.54, 59.87)	0.043^*^	7.22 (1, 39)	0.011^**^	1.94 (1.54, 59.87)	0.162	0.608 (1, 39)	0.440
	14 s	3.69 (1.34, 52.22)	0.048^*^	8.50 (1, 39)	0.006^**^	0.16 (1.34, 52.22)	0.763	0.678 (1, 39)	0.415
	20 s	3.78 (1.61, 62.91)	0.037^*^	4.08 (1, 39)	0.030*	0.40 (1.61, 62.91)	0.626	0.686 (1, 39)	0.413

* *p < 0.05*.

** *p < 0.0167 (Bonferroni-corrected level of significance)*.

*Bold values signify p < 0.05*.

Skin conductance levels showed similar patterns during the reproduction intervals when compared with the respective encoding intervals (Figure 5). Mixed-design ANOVAs confirmed some of the main effects of time to be significant, but again, results were much more ambiguous than during the encoding intervals (Table 3).

#### Slopes of cardiac periods during the encoding intervals of the auditory and visual reproduction tasks

Across groups, cardiac periods increased by 0.09 z-scores per second, 0.05 z-scores per second, and 0.04 z-scores per second during the 8, 14, and 20 s intervals of the auditory reproduction task, respectively. The slopes of cardiac periods did not differ significantly between meditators and controls (Supplementary Table 3).

Cardiac periods increased by 0.08 z-scores per second, 0.04 z-scores per second, and 0.04 z-scores per second during the 8, 14, and 20 s encoding intervals of the visual reproduction task, respectively. Again, the slopes of meditators and controls did not differ (Supplementary Table 3).

#### Slopes of skin conductance levels during the encoding intervals of the auditory and visual reproduction tasks

**Figure 6:**
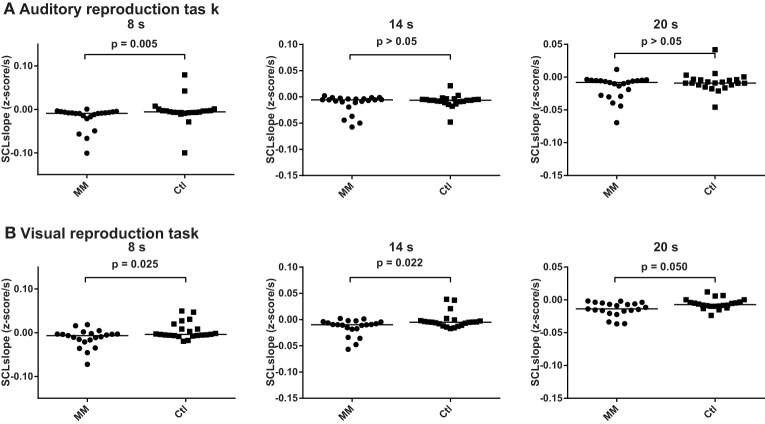
**Statistical dot plots with median (black bars) showing skin conductance slopes during the three encoding intervals of the auditory (A) and visual (B) duration reproduction tasks**. Mann–Whitney *U*-Tests were used to evaluate group differences.

Across groups, skin conductance levels decreased by 0.012 z-scores per second, 0.011 z-scores per second and 0.011 z-scores per second during the 8, 14, and 20 s intervals of the auditory reproduction task, respectively. A significant difference between groups was revealed for the 8-s interval, with a steeper decline in the group of meditators (−0.020 ± 0.026 vs. −0.004 ± 0.020, *p* = 0.005; Figure 6, Supplementary Table 3).

During the visual reproduction task, skin conductance levels decreased across all subjects by 0.004, 0.008 and 0.010 z-scores per second during the 8, 14, and 20 s intervals, respectively. Meditators consistently showed a steeper decline of skin conductance levels than controls (Figure 6, Supplementary Table 3).

### Correlational analyses

Since no differences between groups were revealed the correlational analyses were performed for the whole study sample (*n* = 44).

#### Attentional capacities

Correct answers in the TAP subtest for divided attention correlated positively with heart beat perception scores across all subjects (*r* = 0.381, *p* = 0.011), while the three components of the ANT alerting, orienting, and executive function and ANT reaction times showed no significant association (results not shown). Correct answers in the TAP subtest for divided attention also correlated positively with reproduced duration during the 8 s-encoding interval of the auditory reproduction task (Spearman's *r* = 0.396, *p* = 0.008), and with reproduced duration and reproduction accuracy during the 20 s encoding interval of the visual reproduction task (Spearman's *r* = 0.396, *p* = 0.009; and *r* = 0.390, *p* = 0.010, respectively). Alerting, orienting and executive function in the ANT as well as reaction times did not show such correlations (results not shown).

#### Heart beat perception scores

Heart beat perception scores were not associated with any of the performance variables in the auditory and visual duration estimation tasks, or with the slope of cardiac periods but correlated negatively with the slope of skin conductance levels during the 14 s-encoding interval of the auditory task (Spearman's *r* = -0.404, *p* = 0.009) and with the slope of skin conductance levels during the 8 s-encoding interval of the visual task (Spearman's *r* = −0.451, *p* = 0.003) (Supplementary Table 4).

#### Reproduced duration and duration reproduction accuracy

**Figure 7:**
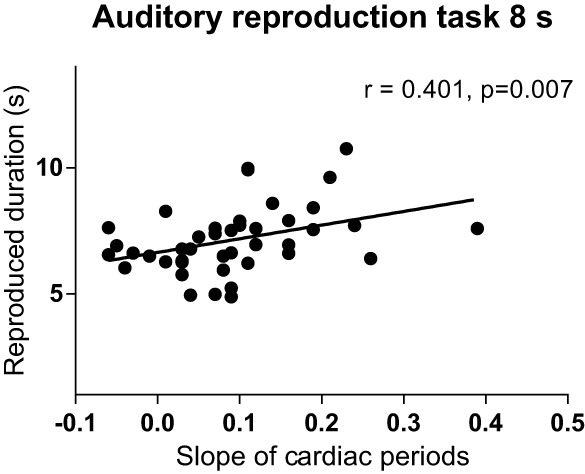
**Relationship between the slope of cardiac intervals and reproduced duration of the 8-s interval of the auditory reproduction task**. *R*, Spearman's correlation coefficient.

During the 8 s-interval of the auditory task, the slopes of cardiac periods correlated positively with reproduced duration (Figure 7; Supplementary Table 5), while the slopes of skin conductance levels during the auditory 8-s intervals showed a negative correlation with duration reproduction accuracy (Spearman's *r* = −0.433, *p* = 0.005; Supplementary Table 6).

## Discussion

We studied the role of attentional capacities, cardiac awareness, and bodily signals for performance in duration reproduction tasks in *n* = 22 experienced mindfulness meditators and *n* = 22 age-, sex-, and education matched controls. Contrary to our hypotheses, results revealed no differences between meditators and controls with respect to performance in duration reproduction abilities, heart beat perception scores, or attentional capacities. Similarly, the slope of cardiac periods did not differ between groups. Meditators, however, showed a steeper decline of skin conductance levels during the 8 s-encoding intervals of both, the visual and auditory duration reproduction task (Figure 5, Table 3), which could be due to higher relaxation abilities in experienced meditators (Steinhubl et al., 2015). However, since we did not find any other group differences, we do not want to over-emphasize this finding. Across measures, we observed large inter-individual differences in both meditators and controls, which should be considered in future research.

Our negative findings have to be interpreted in the way that experienced meditators did not perform more accurately in time perception in the seconds' range. Related, psychophysiological parameters, although in general to some degree associated with time perception in the seconds range (see below), did not differ between the two groups studied. Although mindfulness meditators learn to focus on their bodily sensations, they did not differ from carefully matched controls with regard to interoceptive awareness in the heart beat perception task. Two recent studies have also failed to show that experienced meditators are more accurate in the heart beat perception task (Khalsa et al., 2008; Melloni et al., 2013). Our findings are in contrast to studies showing how self-reported aspects of interoception in daily life are enhanced after extended mindfulness meditation (Bornemann et al., 2014). Moreover, objective indices of judgments related to body sensitivity such as tactile discrimination reveal a better performance after extended body-centered meditation (Fox et al., 2012; Mirams et al., 2013). Expert mindfulness meditators have in general shown to be more sensitive to inner urges and impulses which are important for decision-making and movement initiation (Jo et al., 2014, 2015). The discrepancies with our results may be explained by a dissociation between interoceptive sensibility (i.e., self-attributed interoception) and interoceptive accuracy as assessed in an objective heart beat counting task (Garfinkel et al., 2015): Only self-attributed interoception but not interoceptive accuracy might be enhanced in mindfulness meditators as compared to non-meditating controls. In this context it is important to note that most findings of a relationship between mindfulness meditation and a specific mental function were collected in longitudinal studies. That is, individuals who attended a meditation course or retreat lasting several days or weeks improved performance in attention (Maclean et al., 2010) and timing tasks (Droit-Volet et al., 2015). Most likely, in cross-sectional studies as ours large inter-individual differences not controlled for can mask effects of meditation experience.

Heart beat perception scores were positively related with correct answers in the TAP subtest of divided attention, which confirms previous findings of a relationship between attention to external and internal stimuli (Matthias et al., 2009). Moreover, we found several correlations between heart beat perception scores and performance variables in the duration reproduction tasks, but none of them passed the threshold of significance (Supplementary Table 4). Heart beat perception scores, however, correlated negatively with the slopes of skin conductance levels during the 8 s- and 14 s- interval of the visual and auditory tasks, respectively (Supplementary Table 4). These results suggest that better heart beat perceivers tended to show a steeper decrease of both heart rate and skin conductance levels during the shorter encoding intervals of our tasks. Our results are only partially consistent with results in our previous cohort of younger subjects, who were not selected with respect to meditation experience (Meissner and Wittmann, 2011): In that study, heart beat perception scores were found to correlate positively with duration reproduction accuracy for intervals of 8 and 14 s duration in an identical auditory reproduction task. It should be noted that heart beat perception scores in our present cohort were higher than before [0.78 ± 0.16 SD vs. 0.62 ± 0.18 SD in (Meissner and Wittmann, 2011)]. A ceiling effect may thus explain why we could not reproduce these correlations of our former study. A recent study showed that better heart beat perceivers could better synchronize their heart cycle with the start and stop responses in a time reproduction task with significant correlation for a short 2 s time interval (Pollatos et al., 2014)—a further hint suggesting that interoceptive awareness is indeed associated with better performance in time perception. Again, the heart beat perception score of 0.65 ± 0.19 SD in their study was lower than that in our present cohort.

Further, results revealed that reproduced duration correlated positively with the slope of cardiac periods during the 8-s interval of the auditory task, although only moderately, while duration reproduction accuracy showed a negative correlation with the slopes of skin conductance levels. These results provide further evidence that performance in duration reproduction tasks is linked to changes in autonomic tone during the encoding of duration, namely a shift from sympathetic to parasympathetic dominance (Meissner and Wittmann, 2011; Pollatos et al., 2014). However, in our previous study we found this relationship only for the slopes of cardiac periods and not for the slopes of skin conductance levels, and furthermore for intervals of 14 s and 20 s duration (Meissner and Wittmann, 2011). These discrepancies may be linked to differences in the study sample with respect to age, education, and interoceptive awareness.

In contrast to our previous study, participants performed not only an auditory duration reproduction task but also an otherwise similar duration reproduction task in the visual domain. Remarkably, the analyses of psychophysiological changes confirmed not only our previous finding that cardiac periods increased continuously during the encoding and reproduction intervals of the auditory task (Meissner and Wittmann, 2011), but extend these findings in showing a likewise increase of cardiac periods during the encoding and reproduction intervals of the visual task (Figure 4, Table 2). Similarly, the continuous decrease of skin conductance levels described earlier for the auditory task (Meissner and Wittmann, 2011) could be replicated and was also found during the visual task (Figures 4, 5, Tables 2, 3). As in our former study, linear trends fitted the continuous changes in cardiac periods and skin conductance levels during the encoding of auditory time intervals reasonably well, and the same was true with respect to the visual task (Tables 2, 3). These results suggest that the psychophysiological changes during the encoding of duration are very similar for both auditory and visual stimuli and thus are not modality dependent. That is, an interoceptive mechanism related to time perception may be operating. In both cases, the encoding of duration appears to be accompanied by linear decreases of heart rate and skin conductance levels, indicating a shift from sympathetic to parasympathetic dominance. Furthermore, several relationships of these autonomic changes with both performance and interoceptive awareness scores could be shown. These associations could not be attributed to a stronger attentional load, since attentional capacities, if at all, were found to correlate negatively with the slopes of cardiac periods during the encoding of duration. Thus, even though we found some relationships between attentional capacities and performance in the duration estimation tasks, there was no evidence that the characteristic changes of cardiac periods and skin conductance levels during the encoding of time are related to attentional capacities. Rather, a steady change in autonomic tone, as registered in the brain, could represent a bodily marker for the estimation of duration.

Overall, our results are further indicative of an at least moderate relationship between the experience of time and the heart beat, although we did not find differences between meditators and controls. Recent research has shown how strongly intertwined emotions and feeling states of the body are with the processing of duration (Droit-Volet and Meck, 2007; Dirnberger et al., 2012; Droit-Volet et al., 2013; Wackermann et al., 2014). The association between duration reproduction performance and increasing neural activation in the insular cortex on the one hand and body functions and body experience on the other hand point to the notion that subjective time is embodied (Meissner and Wittmann, 2011; Wittmann, 2013). Body signals may function as an internal reference for subjective time when judging the duration of external events. With this study, we add to findings that may eventually lead to a theory of how we as humans perceive time.

However, it is only fair to state that relative to the overall number of potential correlations across cells (defined by duration, modality, and psychophysiological measures) the found number of correlations cannot be judged as high. That is, one cannot conclude that the specific psychophysiological measures employed (that is, assessing body functions of heart and skin conductance) are sufficient indicators of subjective time. Rather these physiological measures may contribute to the feeling of passing time in a similar way as they are selective indicators of the present condition of the body (Craig, 2015). Subjective time may result or be mediated through the integration of all bodily signals of which we only were able to assess a selection.

### Conflict of interest statement

The authors declare that the research was conducted in the absence of any commercial or financial relationships that could be construed as a potential conflict of interest.
