# Supplementary material for: Psychophysiology of duration estimation in experienced mindfulness meditators and matched controls
Source: Front Psychol. 2015 Aug 18;6:1215. doi: 10.3389/fpsyg.2015.01215 (PMC4539454; doi:10.3389/fpsyg.2015.01215)
Supplement: Supplementary file 1 [file Table1.PDF]

Supplementary Material

Psychophysiology of duration estimation in experienced mindfulness meditators and matched controls

Simone Otten, Eva Schötz, Marc Wittmann, Niko Kohls, Stefan Schmidt, Karin Meissner*

***Correspondence:** Corresponding Author: karin.meissner@med.lmu.de

1.1. Supplementary Tables

Supplementary Table 1. Comparison of attentional capacities between the two study groups.

Variable		Mindfulness Meditators	Matched Controls	p-value[1]
Attention Network Test (ANT)		(n=22)	(n=20)	
	Alerting (ms, mean ± SD)	37.0 ± 18.9	30.9 ± 25.2	0.378
	Orienting (ms, mean ± SD)	45.6 ± 21.3	53.1 ± 26.5	0.322
	Executive function (ms, mean ± SD)	111.8 ± 27.8	120.9 ± 25.4	0.277
	Mean reaction time (ms, mean ± SD)	565.9 ± 61.8	593.8 ± 64.6	0.160
	Mean accuracy (%, mean ± SD)	98.2 ± 1.1	98.6 ± 1.2	0.250[2]
Divided attention (TAP subtest)		(n=22)	(n=22)	
	Correct (mean ± SD)	30.6 ± 1.5	30.2 ± 2.1	0.895[2]
	Incorrect (mean ± SD)	0.8 ± 1.2	1.0 ± 1.3	0.652[2]
	Omissions (mean ± SD)	1.2 ± 1.2	1.4 ± 1.6	0.903[2]

Abbreviations: TAP, Test Battery for Attentional Performance'

[1] t-Test if not otherwise indicated.

[2] Mann-Whitney-U Test.
