# Supplementary material for: Psychophysiology of duration estimation in experienced mindfulness meditators and matched controls
Source: Front Psychol. 2015 Aug 18;6:1215. doi: 10.3389/fpsyg.2015.01215 (PMC4539454; doi:10.3389/fpsyg.2015.01215)
Supplement: Supplementary file 2 [file Table2.PDF]

Supplementary Table 2. Results of the mixed-design ANOVAs for duration reproduction respective accuracy during the three encoding intervals of the auditory and visual duration reproduction tasks. *F*, main effect of group.

	Variable		Mindfulness Meditators (n=22)	Matched Controls (n=22)	*F* (1,42)	p-value
**Auditory Task**	Duration reproduction (s)				0.4	0.514
		8 s	7.3 ± 1.4	7.0 ± 1.3		
		14 s	11.7 ± 2.1	11.2 ± 2.0		
		20 s	16.1 ± 2.7	15.7 ± 3.7		
	Duration reproduction accuracy				0.3	0.588
		8 s	0.78 ± 0.08	0.79 ± 0.09		
		14 s	0.78 ± 0.11	0.77 ± 0.13		
		20 s	0.77 ± 0.11	0.72 ± 0.14		
**Visual Task**	Duration reproduction (s)				0.9	0.345
		8 s	7.8 ± 2.0	8.2 ± 1.7		
		14 s	12.4 ± 2.3	13.1 ± 2.1		
		20 s	16.3 ± 2.7	16.8 ± 2.4		
	Duration reproduction accuracy				0.4	0.537
		8 s	0.75 ± 0.14	0.76 ± 0.09		
		14 s	0.79 ± 0.12	0.81 ± 0.10		
		20 s	0.77 ± 0.11	0.79 ± 0.09		
