# Supplementary material for: Psychophysiology of duration estimation in experienced mindfulness meditators and matched controls
Source: Front Psychol. 2015 Aug 18;6:1215. doi: 10.3389/fpsyg.2015.01215 (PMC4539454; doi:10.3389/fpsyg.2015.01215)
Supplement: Supplementary file 3 [file Table3.PDF]

Supplementary Table 3: Group differences in slopes of cardiac periods and of skin conductance levels during the three encoding intervals of the auditory and visual duration reproduction tasks.

		Variable	Mindfulness Meditators	Matched Controls	p-value[1]
**Slopes of cardiac periods**	**Auditory**	N	22	22	
		8 s	0.073 ± 0.082	0.102 ± 0.102	0.299
		14 s	0.043 ± 0.031	0.049 ± 0.042	0.575
		20 s	0.032 ± 0.030	0.042 ± 0.032	0.294
	**Visual**	N	20	22	
		8 s	0.073 ± 0.072	0.085 ± 0.095	0.649
		14 s	0.038 ± 0.034	0.049 ± 0.032	0.263
		20 s	0.050 ± 0.027	0.036 ± 0.027	0.088
**Slopes of skin conductance levels**	**Auditory**	N	21	20	
		8 s	-0.020 ± 0.026	-0.004 ± 0.032	**0.005**[2][**]
		14 s	-0.014 ± 0.018	-0.008 ± 0.012	0.938[2]
		20 s	-0.016 ± 0.018	-0.008 ± 0.016	0.481[2]
	**Visual**	N	21	20	
		8 s	-0.012 ± 0.021	0.005 ± 0.020	**0.025**[2][*]
		14 s	-0.015 ± 0.016	-0.001 ± 0.015	**0.022**[2][*]
		20 s	-0.014 ± 0.011	-0.006 ± 0.008	0.050[2]

[1] *t*-Test if not otherwise indicated.

[2] Mann-Whitney-U Test.

[*] $p<0.05$.

[**] $p<0.0167$ (Bonferroni-corrected level of significance).
