# Supplementary material for: Psychophysiology of duration estimation in experienced mindfulness meditators and matched controls
Source: Front Psychol. 2015 Aug 18;6:1215. doi: 10.3389/fpsyg.2015.01215 (PMC4539454; doi:10.3389/fpsyg.2015.01215)
Supplement: Supplementary file 4 [file Table4.PDF]

Supplementary Table 4: Spearman-Rho correlations for heart beat perception scores with reproduced duration, duration reproduction accuracy, slopes of cardiac periods and slopes of sin conductance levels in the total group

	**Auditory**			**Visual**		
**Variable**	**R**	**N**	**p-value**	**R**	**N**	**p-value**
Reproduced duration						
8 s	0.037	44	0.814	-0.028	44	0.859
14 s	0.056	44	0.719	0.090	44	0.563
20 s	0.005	44	0.973	0.134	44	0.385
Duration reproduction accuracy						
8 s	0.286	44	0.060	0.291	44	0.055
14 s	0.194	44	0.208	0.285	43	0.064
20 s	0.159	44	0.302	0.267	43	0.083
Slopes of cardiac periods						
8 s	0.042	44	0.785	0.263	42	0.092
14 s	0.198	44	0.199	0.116	42	0.463
20 s	0.280	44	0.066	0.127	42	0.424
Slopes of skin conductance levels						
8 s	-0.244	41	0.125	**-0.451**	**41**	**0.003****
14 s	**-0.404**	**41**	**0.009****	0.052	41	0.748
20 s	-0.297	41	0.060	-0.244	41	0.125

$^{*}p<0.05$

** $p<0.0167$ (Bonferroni-corrected level of significance).
