# Supplementary material for: Psychophysiology of duration estimation in experienced mindfulness meditators and matched controls
Source: Front Psychol. 2015 Aug 18;6:1215. doi: 10.3389/fpsyg.2015.01215 (PMC4539454; doi:10.3389/fpsyg.2015.01215)
Supplement: Supplementary file 5 [file Table5.PDF]

Supplementary Table 5: Spearman-Rho correlations for slopes of cardiac periods and skin conductance levels with reproduced duration in the total group

		Auditory			Visual		
**Variable**		**R***	**N**	**p-value**	**R***	**N**	**p-value**
Slopes of cardiac periods							
	8 s	**0.401**	**44**	**0.007****	0.011	42	0.951
	14 s	0.099	44	0.523	-0.038	42	0.809
	20 s	-0.179	44	0.246	-0.086	42	0.587
Slopes of skin conductance levels							
	8 s	-0.013	41	0.936	0.044	40	0.784
	14 s	0.096	41	0.552	0.297	41	0.059
	20 s	-0.020	41	0.900	0.234	41	0.141

*p<0.05

** p<0.0167 (Bonferroni-corrected level of significance).
