# Supplementary material for: Psychophysiology of duration estimation in experienced mindfulness meditators and matched controls
Source: Front Psychol. 2015 Aug 18;6:1215. doi: 10.3389/fpsyg.2015.01215 (PMC4539454; doi:10.3389/fpsyg.2015.01215)
Supplement: Supplementary file 6 [file Table6.PDF]

Supplementary Table 6: Spearman-Rho correlations for slopes of cardiac periods and skin conductance levels with duration reproduction accuracy in the total group

		Auditory			Visual		
**Variable**		**R***	**N**	**p-value**	**R***	**N**	**p-value**
Slopes of cardiac periods							
	8 s	-0.014	44	0.930	0.111	42	0.484
	14 s	0.144	44	0.350	0.126	42	0.425
	20 s	-0.014	44	0.927	-0.032	42	0.839
Slopes of skin conductance levels							
	8 s	**-0.433**	**41**	**0.005****	-0.272	41	0.085
	14 s	-0.083	41	0.606	-0.014	41	0.929
	20 s	-0.168	41	0.293	0.026	41	0.874

*p<0.05

** p<0.0167 (Bonferroni-corrected level of significance).
